# Development and Customization of a Color-Coded Microbeads-Based Assay for Drug Resistance in HIV-1 Reverse Transcriptase

**DOI:** 10.1371/journal.pone.0109823

**Published:** 2014-10-14

**Authors:** Lijun Gu, Ai Kawana-Tachikawa, Teiichiro Shiino, Hitomi Nakamura, Michiko Koga, Tadashi Kikuchi, Eisuke Adachi, Tomohiko Koibuchi, Takaomi Ishida, George F. Gao, Masaki Matsushita, Wataru Sugiura, Aikichi Iwamoto, Noriaki Hosoya

**Affiliations:** 1 Research Center for Asian Infectious Diseases, the Institute of Medical Science, the University of Tokyo, Tokyo, Japan; 2 Japan-China Joint Laboratory of Molecular Immunology and Molecular Microbiology, Institute of Microbiology, Chinese Academy of Sciences, Beijing, P. R. China; 3 Division of Infectious Diseases, Advanced Clinical Research Center, the Institute of Medical Science, the University of Tokyo, Tokyo, Japan; 4 AIDS Research Center, National Institute of Infectious Diseases, Tokyo, Japan; 5 Department of Infectious Disease Control, International Research Center for Infectious Diseases, the Institute of Medical Science, the University of Tokyo, Tokyo, Japan; 6 Division of Infectious Diseases and Applied Immunology, Research Hospital, The Institute of Medical Science, The University of Tokyo, Tokyo, Japan; 7 CAS Key Laboratory of Pathogenic Microbiology and Immunology, Institute of Microbiology, Chinese Academy of Sciences, Beijing, P. R. China; 8 Biotech Research and Development, Wakunaga Pharmaceutical Corporation, Hiroshima, Japan; University of Pittsburgh, United States of America

## Abstract

**Background:**

Drug resistance (DR) of HIV-1 can be examined genotypically or phenotypically. Although sequencing is the gold standard of the genotypic resistance testing (GRT), high-throughput GRT targeted to the codons responsible for DR may be more appropriate for epidemiological studies and public health research.

**Methods:**

We used a Japanese database to design and synthesize sequence-specific oligonucleotide probes (SSOP) for the detection of wild-type sequences and 6 DR mutations in the clade B HIV-1 reverse transcriptase region. We coupled SSOP to microbeads of the Luminex 100 xMAP system and developed a GRT based on the polymerase chain reaction (PCR)-SSOP-Luminex method.

**Results:**

Sixteen oligoprobes for discriminating DR mutations from wild-type sequences at 6 loci were designed and synthesized, and their sensitivity and specificity were confirmed using isogenic plasmids. The PCR-SSOP-Luminex DR assay was then compared to direct sequencing using 74 plasma specimens from treatment-naïve patients or those on failing treatment. In the majority of specimens, the results of the PCR-SSOP-Luminex DR assay were concordant with sequencing results: 62/74 (83.8%) for M41, 43/74 (58.1%) for K65, 70/74 (94.6%) for K70, 55/73 (75.3%) for K103, 63/73 (86.3%) for M184 and 68/73 (93.2%) for T215. There were a number of specimens without any positive signals, especially for K65. The nucleotide position of A2723G, A2747G and C2750T were frequent polymorphisms for the wild-type amino acids K65, K66 and D67, respectively, and 14 specimens had the D67N mutation encoded by G2748A. We synthesized 14 additional oligoprobes for K65, and the sensitivity for K65 loci improved from 43/74 (58.1%) to 68/74 (91.9%).

**Conclusions:**

We developed a rapid high-throughput assay for clade B HIV-1 DR mutations, which could be customized by synthesizing oligoprobes suitable for the circulating viruses. The assay could be a useful tool especially for public health research in both resource-rich and resource-limited settings.

## Introduction

Since combination antiretroviral therapy (cART) was introduced, the prognosis of patients with HIV-1 infection has improved dramatically [1], [2]. In resource-rich settings, new classes, new drugs or new formulations of previously-known classes of antiretroviral drugs (ARV) have been introduced continuously for clinical use. Nucleoside/nucleotide reverse transcriptase inhibitor (NRTI) resistance has declined over time in resource-rich settings, presumably reflecting the improvement of treatment regimens [3], [4]. Rates of transmitted HIV-1 drug resistance (DR) have remained limited also in resource-limited settings; however, limitation of the first-line and subsequent regimens would be a concern. cART consisting of two NRTIs and one non-nucleoside reverse transcriptase inhibitors (NNRTI), most often zidovudine (AZT) + lamivudine (3TC) or stavudine (d4T) + 3TC plus nevirapine (NVP) or efavirenz (EFV), has been widely used as the treatment regimen in the resource-limited settings [5], [6]; consequently, DR might become a larger public health challenge in the developing countries.

DR can be examined genotypically or phenotypically [7] (http://www.aidsmap.com/pdf/Resistance-tests/page/1044559/). Although sequencing is the gold standard of the genotypic resistance testing (GRT), high-throughput GRT targeted to the codons responsible for DR may be more convenient and suitable for public health research [8], [9]. We applied the PCR-SSOP-Luminex method [10]–[12] to an HIV-1 GRT. As an initial approach, we focused on designing an assay for six major DR mutations: M41L, K65R, K70R, K103N, M184V and T215Y/F. M41L, K70R, T215F/Y are examples of thymidine analogue mutations (TAMs) and associated with AZT and d4T [13] (HIV Drug resistance database, Stanford University, http://hivdb.stanford.edu/index.html). K65R is associated multi-nucleoside and nucleotide DR. Although K65R is selected by nucleotide reverse transcriptase inhibitor tenofovir (TDF) usually, it can be selected by d4T. K103N is highly associated with EFV and NVP resistance. The K103N mutation reduces susceptibility to NVP by 50-flod, and EFV by 20-fold. M184V is highly associated with 3TC and emtricitabine (FTC) resistance, and reduce the susceptibility to 3TC by 200-fold. The monitoring of these six DR mutations should be important for molecular epidemiologic study estimating the efficacy of anti-HIV drugs especially in resource limited settings. We synthesized the oligonucleotides for the primers and probes based on the Japanese data base on reverse transcriptase mutations. In order to validate the initial assay system and examine the flexibility for customization, we focused on the clade B HIV-1 which is most prevalent in Japan. Here we report the results of the comparison between sequencing and the PCR-SSOP-Luminex assay using the specimens of a Japanese cohort.

## Methods

### PCR-SSOP-Luminex assay

HIV-1 DR genotyping described here is based on the reverse SSOP method coupled with a microsphere beads array platform (Luminex Corporation, Austin, TX, USA). Briefly, the method involves PCR amplification by biotinylated primers, hybridization to nucleotide probes coupled to microbeads, detection of the bound PCR products by streptavidin-phycoerythrin (SAPE) reaction, and quantitation by measurement of median fluorescence intensity (MFI).

Color-coded microbeads were coupled to oligoprobes derived from DR mutations or conserved sequences in HIV-1 RT coding region. These synthesized probes were modified at the 5′-end with a terminal amino group and covalently bound to the carboxylated fluorescent microbeads using ethylene dichloride (EDC), following the procedures recommended by the manufacturer (Wakunaga Pharmaceutical Co. Ltd, Hiroshima, Japan). Briefly, 6.25×10^5^ carboxylated microbeads were suspended in 50 µl of 0.1 M MES (2-(N-morpholino) ethane sulfonic acid, pH 4.5 (Dojindo Laboratories, Kumamoto Techno Research Park, Kumamoto, Japan). Afterwards, 0.5 µM of amine-substituted oligonucleotide probes was added, followed by 100 mg/ml EDC (1-Ethyl-3-(3-dimethylaminopropyl) carbodiimido hydrochloride) (Pierce Biotechnology, Rockford, IL, USA), and the mixture was incubated in the dark for 30 min at 25°C. The EDC addition and incubation were repeated twice and the microbeads were washed once with 0.02% Tween-20 and once with 0.1% SDS. After the final wash, the pellets were resuspended in 50 µl TE buffer (pH 8.0), and counted on a hemocytometer. The concentration of fluorescence-labeled microbeads coupled to oligonucleotide probes (oligobeads) was adjusted to 8000–12000/µl, and oligobeads were stored at 4°C in the dark.

Five-microliter aliquots of the 5′-biotinlabeled amplified DNA were added to wells in a 96-well PCR tray containing 5 µl/well of denaturation solution, and allowed to denature for 5 min at RT. Hybridization mixture was prepared using oligobeads stocks, SAPE and hybridization solution, according to the manufacturer’s instructions (Wakunaga Pharmaceutical Co. Ltd, Hiroshima, Japan). Twenty-five-microliter aliquots of hybridization mixture containing 500 each sequence-specific oligobeads were added to each well. The amplicons were hybridized at 55°C for 30 min using the thermal cycler. Hybridized amplicons were washed twice with 75 µl of wash solution in each well. Reaction outcomes were measured by the Luminex 100 flow cytometer that is equipped with two types of lasers. The bead populations were detected and identified using the 635 nm laser. The SAPE fluorescence of the biotin labeled amplicons that had hybridized to the oligobeads was quantitated using the 532 nm laser. The MFI of SAPE was used to quantify the amount of DNA bound to the oligobeads. Assays were performed in triplicate.

### Site-directed mutagenesis and plasmid construction

To assess the feasibility of the assay system, we chose cloned SF2 genome (GenBank accession number K02007) as a template. Synthesized PCR fragments with DR mutations created by site-directed mutagenesis were inserted into the SF2 genome. The numbering system used to refer to DR mutations was based on HXB2 genome (HXB2 location 2485–3309, GenBank accession number K03455) sequences. The SF2 and HXB2 genome had the same sequence in the regions covered by the probes.

**Figure 1:**
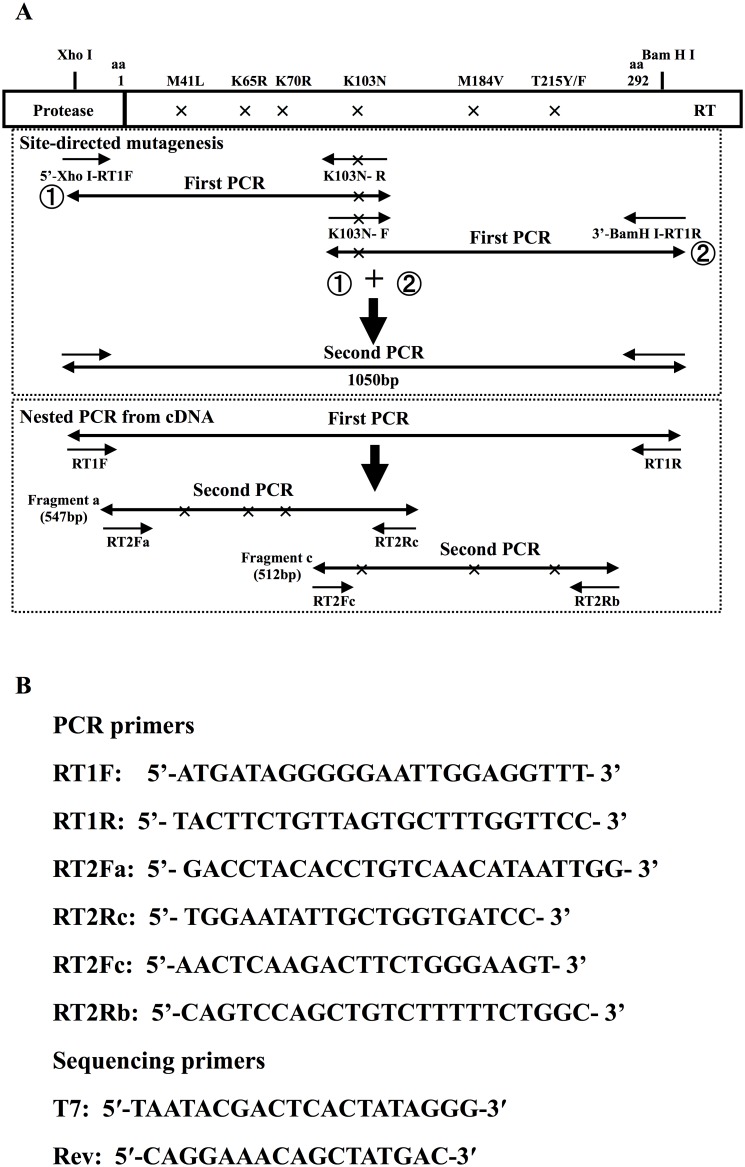
Schematic representation of PCR amplification and sequences of primers for PCR and sequencing. (A) Top: Site-directed mutagenesis using oligonucleotide is shown using K103N as an example. Desired mutations in the reverse transcriptase gene were engineered in two PCR fragments, then incorporated into a larger fragment (1050 bp, HXB2 location 2388–3425) by the second PCR, and cloned into pBluescript II SK (+) at *Xho*I-*BamH*I sites. Bottom: Negative strand cDNA was synthesized from patients’ plasma. After the first strand PCR using RT1F and RT1R as primers, Fragment a or Fragment c were amplified by nested PCR. (B) Primer sequences for PCR amplification and sequencing.

Plasmid p9B/R7 from the HIV-1 SF2 strain was a kind gift of Dr. T. Shioda (Osaka University, Osaka, Japan) [14], [15]. An *Xho* I- *BamH* I fragment including the pol gene from p9B/R7 was subcloned into pBluescript II SK (+) (Stratagene, La Jolla, CA, USA). As shown in Fig. 1A, various DR mutations were introduced into the *Xh*o I- *BamH* I fragment of the plasmid by site-directed mutagenesis using oligonucleotides and PCR methods as previously described [16], and were confirmed by sequencing.

### Clinical specimens

Sixty subjects were selected from among patients participating in an ongoing study on microbes at an HIV outpatient clinic affiliated with the Institute of Medical Science, the University of Tokyo (IMSUT). The study was approved by the internal review board of IMSUT (20-31-1120), and all subjects provided written informed consent. The median HIV-1 RNA and CD4 cells count at sampling were 4.15 (range 2.60–5.88) log 10 copies/ml and 264 (range: 9–902) cells/µl, respectively. Seventy-four specimens from 60 patients were analyzed in this study. Forty-eight patients contributed one plasma specimen, 10 patients contributed two plasma specimens from different time points, and two patients contributed three separate plasma specimens. Twenty-two specimens were from patients who were treatment-naïve when the plasma specimens were obtained. The remaining 52 specimens were from patients on failing treatment including NRTI at the time of sample collection.

### Viral RNA extraction, cDNA synthesis, and PCR amplification

Viral RNA was extracted from 140 µl plasma using QIAamp Viral RNA Mini Kit (QIAGEN, Valencia, CA, USA) and eluted with 60 µl AVE buffer. For cDNA synthesis, 55 µl of RNA solution was mixed with 5 µl of 100 pmol/µl random primer (TaKaRa Bio, Otsu, Shiga, Japan) or specific primer, RT1R (Fig. 1B), and 5 µl of 10 mM dNTPs, and denatured at 70°C for 10 min. The RT mixture containing 20 µl of 5× First-Strand buffer, 5 µl of 0.1 M DTT, 5 µl of RNaseOUT Recombinant RNase inhibitor (40 U/µl; Invitrogen, Carlsbad, California, USA) and 5 µl of SuperScript III RT (200 U/µl, Invitrogen) was added to the 65 µl denatured viral RNA-primer-dNTP mixture. The reaction mixture (100 µl final volume) was incubated at 25°C for 5 min for annealing and then at 55°C for 60 min for reverse transcription. The reaction was inactivated by heating at 70°C for 15 min.

RT gene fragments were amplified by nested PCR from cDNAs or by single PCR from plasmids. For the first reaction a 1050 bp fragment from the RT coding region was amplified from 5 µl aliquots of cDNAs using RT1F and RT1R as outer primers in a reaction mixture containing 50 µl of 1×Prime STAR buffer, 0.2 mM of each dNTPs, 0.5 µM of each primer, and 0.5 µl Prime STAR HS DNA Polymerase (25 U/µl, TaKaRa Bio, Otsu, Shiga, Japan). Amplification conditions consisted of 35 cycles denaturation at 98°C for 10 s, annealing at 55°C for 5 s, and extension at 72°C for 30 s.

For the second reaction of the nested PCR and for the single PCR from plasmids (0.1 pg), RT coding fragments were amplified in two PCR fragments using two 5′ biotinylated primer sets, PS1 and PS2, as described previously [17]. The PS1 primer set produced a 547 bp amplicon that was used to detect M41L, K65R, K70R, and K103N, and the PS2 primer set produced a 512 bp amplicon that was used to detect K103N, M184V, and T215Y/F (Fig. 1A). The reaction mixture used 5 µl of the first PCR products or 0.1 pg of plasmid DNA in a final volume of 50 µl, as described above, with amplification conditions as follows: 25 cycles (second nested PCR) or 35 cycles (plasmid DNA amplification) of denaturation at 98°C for 10 s, annealing at 55°C for 5 s, and extension at 72°C for 1 min. The PCR amplicons were used for Luminex detection or direct sequencing.

### Sequencing

PCR products were purified with the QIAquick PCR Purification Kit (QIAGEN, Valencia, CA, USA) and were directly sequenced in both directions using ABI 3130xl genetic analyzer (Applied Biosystems, Foster City, CA, USA) and Big Dye terminator V3.1 cycle sequencing kit (Applied Biosystems). In the case of sequence ambiguity due to a coexistence of multiple nucleotides, we confirmed the sequence by cloning and sequencing. For cloning and sequencing, the purified PCR fragments were phosphorylated using T4 polynucleotide kinase (TaKaRa Bio, Otsu, Shiga, Japan) and inserted into the dephosphorylated *EcoR*V restriction site of pBluescript II SK(+). Inserts were sequenced using T7 and Rev universal primers (Fig. 1B).

### HIV-1 Japanese surveillance database

In Japan 10 university hospitals, 5 medical centers, 5 public health laboratories, and the National Institute of Infectious Diseases are collaborating in the surveillance of newly diagnosed HIV/AIDS cases. HIV/AIDS patients with both acute and chronic infections, newly diagnosed at these centers since January 2003 were enrolled [18]. Prevalence of DR codons in these patients was determined by analysis of sequences of clade B HIV-1 reverse transcriptase positions 1–240 amino acids.

### Statistical analysis

GraphPad Prism 5.0 software (GraphPad Software Inc., San Diego, CA) was used for statistical data analysis. Statistical significance was defined as P<0.05.

## Results

### Design of oligoprobes based on the database of clade B HIV-1 sequences in Japan

Based on the frequency of the codon usage in the Japanese surveillance database for amino acids M41, K65, K70, K103, M184 and T215 in RT gene, we designed the nucleotide sequence of 18 oligoprobes for DR mutation and 5 standard probes (Table 1). We adopted nucleotide sequences of HXB2 strain for the flanking sequences. Synthesized oligoprobes could cover 71.2%, 59.3%, 80.8%, 71.1%, 85.7% and 71.7% of M41, K65, K70, K103, M184 and T215, respectively (Table 1). Five standard probes were designed in the conserved region of RT gene and used as the assay control.

**Table 1:** Design of oligoprobes based on clade B HIV-1 sequences from Japanese surveillance database.

Locus	geneticcode	Frequency(%)	Name of probe	Oligoprobes	Nucleotide sequence(5'-3')	100% match isolates ofdatabase
M41					TGT ACA GAA ATG GAA	
	M41-ATG	795/795(100)	M41M-ATG	GTACAGAAATGGAA	— --- --- --- ---	618/868(71.2%)
	M41L-TTG	109/180(60.6)	M41L-TTG	GTACAGAATTGGAA	— --- --- T— ---	
	M41L-CTG	69/180(38.3)	M41L-CTG	GTACAGAACTGGAA	— --- --- C— ---	
	M41L-TTA	1/180(0.6)				
	M41L-TTR	1/180(0.6)				
K65					ATA AAG AAA AAA GAC AGT	
	K65-AAA	961/1012(95.0)	K65K-AAA	ATAAAGAAAAAAGACAG	--- --- --- --- --- —	516/870(59.3%)
	K65-AAG	30/1012(3.0)				
	K65-AAR	21/1012(2.1)				
	K65R-AGA	7/7(100)	K65R-AGA	ATAAAGAGAAAAGACAG	--- --- -G- --- --- —	
	K65R-AGG	0/7(0.0)				
K70					GAC AGT ACT AAA TGG AGA	
	K70-AAA	849/885(95.9)	K70K-AAA-1	GACAGTACTAAATG	--- --- --- --- —	621/769(80.8%)
			K70K-AAA-2	GTACTAAATGGAGA	--- --- --- --- ---	
	K70-AAG	17/885(1.9)				
	K70-AAR	19/885(2.1)				
	K70R-AGA	85/88(96.6)	K70R-AGA	GTACTAGATGGAGA	--- --- -G- --- ---	
	K70R-AGG	0/88(0.0)	K70R-AGG	GTACTAGGTGGAGA	--- --- -GG --- ---	
	K70R-AGR	3/88(3.4)				
K103					TTA AAA AAG AAA AAA TCA GTA	
	K103-AAA	815/867(94.0)	K103K-AAA	AAAAAAGAAAAAATCAG	- --- --- --- --- --- -	558/785(71.1%)
	K103-AAG	25/867(2.9)				
	K103-AAR	27/867(3.1)				
	K103N-AAC	112/128(87.5)	K103N-AAC	AAAAAAGAACAAATCAG	- --- --- —C --- --- -	
	K103N-AAT	3/128(2.3)	K103N-AAT	AAAAAAGAATAAATCAGT	- --- --- —T --- --- —	
	K103N-AAY	13/128(10.2)				
M184					TAT CAA TAC ATG GAT GAT	
	M184-ATG	659/659(100)	M184M-ATG	TATCAATACATGGATG	--- --- --- --- --- -	761/888(85.7%)
	M184V-GTG	295/335(88.1)	M184V-GTG	TATCAATACGTGGATG	--- --- --- G— --- -	
	M184V-GTA	14/335(4.2)				
	M184V-GTR	26/335(7.8)				
T215					TGG GGA TTT ACC ACA CCA	
	T215-ACC	692/724(95.6)	T215T-ACC-1	GGGGATTTACCACA	— --- --- --- ---	583/813(71.7%)
			T215T-ACC-2	GGGGATTTACCACACCA	— --- --- --- --- ---	
	T215-ACT	13/724(1.8)				
	T215-ACA	7/724(1.0)				
	T215-ACG	3/724(0.4)				
	T215-ACY	8/724(1.1)				
	T215-ACM	1/724(0.1)				
	T215F-TTC	54/54(100)	T215F-TTC	GGGGATTTTTCACAC	— --- --- TT- ---	
	T215Y-TAC	182/185(98.4)	T215Y-TAC	GGGGATTTTACACAC	— --- --- TA- --- -	
	T215Y-TAT	2/185(1.1)				
	T215Y-TAY	1/185(0.5)				
Standardprobes			S2582	ATTAAAGCCAGGAATGGAT		
			S2693	AAAAATTGGGCCTGAAAAT		
			S3230	CCTTTGGATGGGTTATGAA		
			S3252	CATCCTGATAAATGGACAG		
			S3243	TATGAACTCCATCCTGA		

R: Mixed base of A and G; Y: Mixed base of C and T; M: Mixed base of C and A.

### Evaluation of PCR-SSOP-Luminex DR assay using cloned HIV-1

**Figure 2:**
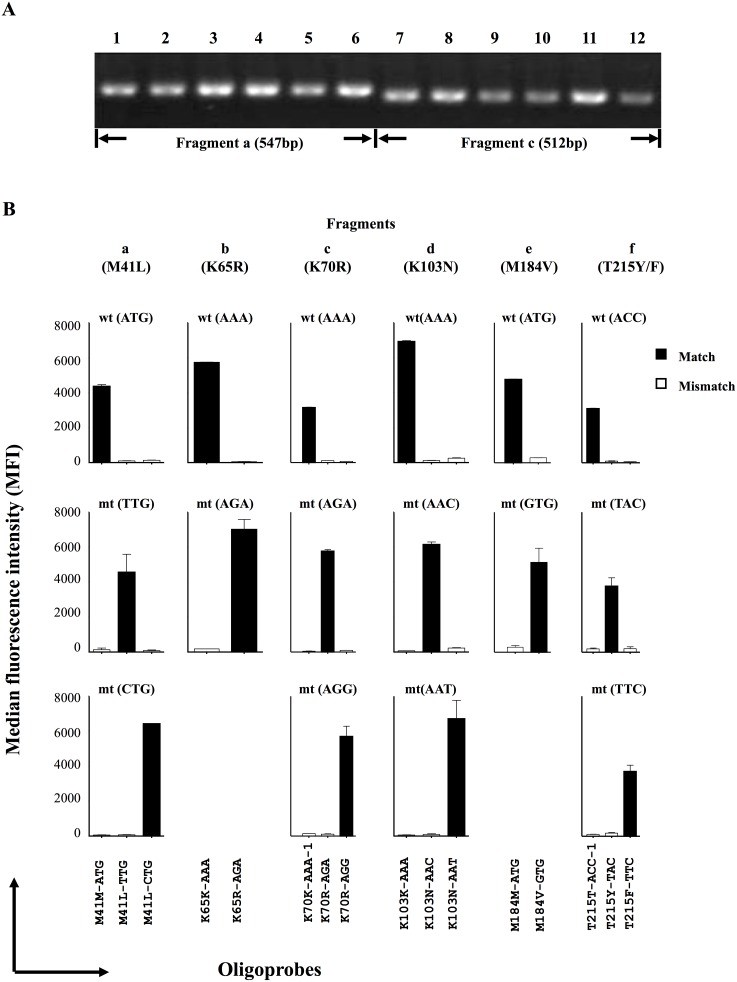
Validation of PCR-SSOP-Luminex assay using plasmids as templates. (A) Agarose gel electrophoresis of amplified fragments. Lanes 1–6: Fragment a (547 bp). Lanes 7–14: Fragment c (512 bp). 1, wild type; 2, M41L-TTG; 3, M41L-CTG; 4, K65R-AGA; 5, 70R-AGA; 6, 70R-AGG; 7, wild type; 8, 103N-AAC; 9, K103N-AAT; 10, M184V-GTG; 11, T215Y-TAC; 12, T215F-TTC. (B) Median fluorescence intensities (MFIs). The plasmid in the test sample is indicated on the top of each panel. Oligoprobes used for detection are indicated at the bottom. Matched results are shown as black bars, mismatched results as white bars. Assays were performed in triplicate. The mean ± standard deviation is shown at the top of each bar.

We examined the sensitivity and specificity of the PCR-SSOP-Luminex DR assay using cloned HIV-1. The test fragments were amplified (Fig. 2A), and the amplicons were hybridized to the 16 oligobeads. We performed three independent assays with triplicate hybridization and detection in each assay. The mean positive signal and standard deviation were 5237±1398 (Fig. 2B). The CV% of positive signal and standard deviation were 10.1% ±10.7. The mean of negative signal and standard deviation were 131.2±69.4. The CV% of negative signal and standard deviation were 21.6±23.6 (mean ± S.D.). MFIs of hybridization signals were clearly high only when the oligoprobes matched the mutations in the fragments (Fig. 2B). These data confirmed that the assay system could discriminate one base mismatch at M41, K65, K70, K103, M184 and T215 codons in the plasmid-probe model system.

**Figure 3:**
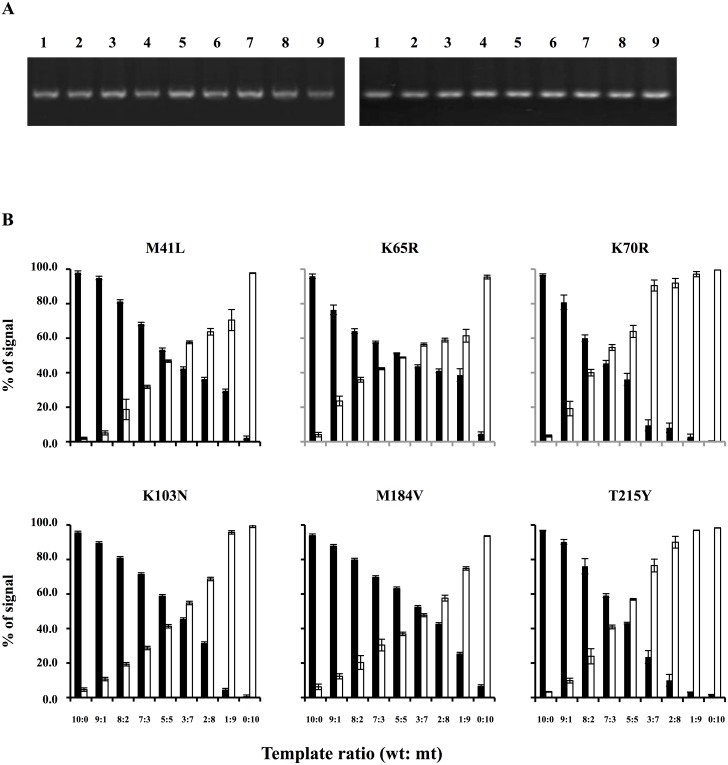
Assay sensitivity in a mixture. (A) Agarose gel electrophoresis of mixtures of amplified fragments. Left panel: Fragment a (547 bp). Right panel: Fragment c (512 bp). Wild-type:mutant ratio; Lanes 1 (10∶0); 2 (9∶1); 3 (8∶2); 4 (7∶3); 5 (5∶5); 6 (3∶7); 7 (2∶8); 8 (1∶9); 9 (0∶10). (B) Signals from wild type probes (black bars) and mutant probes (white bars) in each mixture. “% of signal” was calculated by “MFI of wild type or mutant signal” divided by “MFI of wild type plus mutant signal” and multiplied by 100. Triplicate experiments were performed three times. “% of signal” is shown with standard deviations.

Next, we examined the sensitivity to detect a particular sequence in a mixture for each DR-related site. The plasmids carrying the wild type and mutant sequences were mixed at various ratios. In samples containing only the wild type sequences, the mean background signal (% ±2SD) was 2.0% ±1.2, 4.1% ±2.6, 3.3% ±1.2, 4.6% ±1.8, 6.2% ±3.2 and 3.3% ±0.4 at M41, K65, K70, K103, M184 and T215, respectively (Fig. 3B). The signal for the mutant was judged positive when “% signal” from the mutant oligobeads exceeded the mean + 2SD; 3.2%, 6.7%, 4.5%, 6.4%, 9.4%, 3.7% at M41, K65, K70, K103, M184 and T215, respectively. Actual signals from the mutant oligoprobes at 9∶1 (wild type:mutant) mixture at these sites was 5.1%, 23.6%, 19.2%, 10.7%, 12.3%, 9.8%, respectively. Therefore, we infer that the assay can detect 10% DR mutants in the population. There was a big variation (5.1 to 23.6%) in detection of 10% mixtures. We suppose that the difference in minor variant detection could be caused by the melting temperature (GC content and length). Although we compared the GC content and probe length, we could not find a reasonable explanation from this list (data not shown).

### Identification of DR mutations in clinical specimens by sequencing

In order to determine the sequence, we amplified the RT gene in two separate fragments from frozen plasma (Fig. 1A). We succeeded to amplify fragment a (containing M41, K65, K70) from all 74 specimens, but failed to amplify fragment c (containing K103, M184, T215) in one patient. Infection with clade B HIV-1 was confirmed by phylogenetic analysis of the RT gene. DR mutations were found at codons M41L (n = 22), K65R (n = 3), K70R (n = 10), K103N (n = 7), M184V (n = 21) and T215Y/F (n = 22) in 40 specimens (Table 2).

**Table 2:** Comparison of the results between sequencing and PCR-SSOP-Luminex assay.

Position	Aminoacid	Codons	Number of specimens (percentage)	
			Direct sequencing	PCR-SSOP-Luminex
41	Met	ATG	52 (70.3)	41 (55.4)
	Leu	TTG	16 (21.6)	16 (21.6)
		CTG	5 (6.8)	5 (6.8)
	Met, Leu mix		1 (1.4)	0
	No reaction		-	12 (16.2)
65	Lys	AAA	64 (86.5)	41 (55.4)
		AAG	4 (5.4)	0
		AAA, AAG mix	3 (4.1)	2 (2.7)
	Arg	AGA	3 (4.1)	0
	No reaction		-	31 (41.9)
70	Lys	AAA	62 (83.8)	61 (82.4)
		AAG	2 (2.7)	0
	Arg	AGA	8 (10.8)	8 (10.8)
		AGG	1 (1.4)	1 (1.4)
	Lys, Arg mix		1 (1.4)	0
	No reaction		-	4 (5.4)
103	Lys	AAA	60 (82.2)	50 (68.5)
		AAG	1 (1.4)	0
		AAA, AAG mix	1 (1.4)	0
	Asn	AAC	4 (5.5)	2 (2.7)
		AAT	2 (2.7)	2 (2.7)
		AAC, AAT mix	1 (1.4)	1 (1.4)
	Arg	AGA	4 (5.5)	0
	No reaction		-	18 (24.7)
184	Met	ATG	52 (71.2)	46 (63.0)
	Val	GTG	19 (26.0)	16 (21.9)
		GTA	1 (1.4)	0
	Met, Val mix		1 (1.4)	1 (1.4)
	No reaction		-	10 (13.7)
215	Thr	ACC	48 (65.8)	46 (63.0)
	Tyr	TAC	16 (21.9)	15 (20.5)
	Phe	TTC	6 (8.2)	6 (8.2)
	Thr, Tyr mix		1 (1.4)	1 (1.4)
	Other		2 (2.7)	0
	No reaction		-	5 (6.8)

### Identification of DR mutations in clinical specimens by the PCR-SSOP-Luminex DR assay

**Figure 4:**
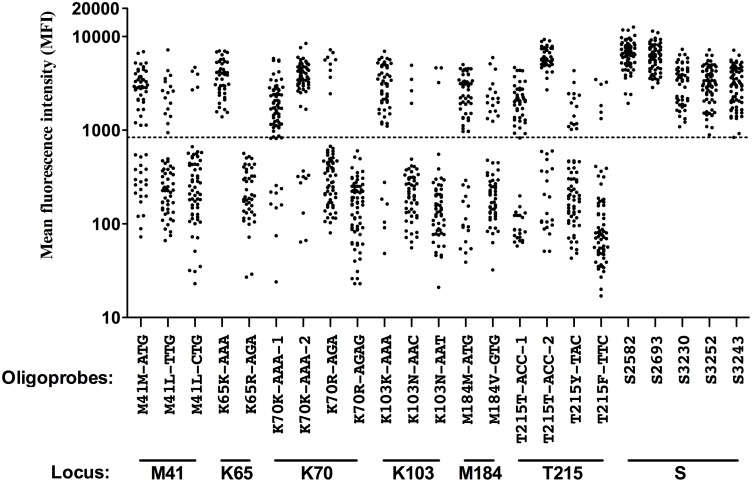
PCR-SSOP-Luminex DR assay of clinical samples. Results of 18 probes for 6 DR loci and 5 standard probes are shown. Each dot represents the mean of triplicates. Dashed line indicates the cut-off value, the lowest MFI among 5 standard probes (MFI = 837.5).

We performed the PCR-SSOP-Luminex DR assay on 74 specimens whose DR mutations had been sequenced (Fig. 4). The lowest MFI of five standard probes (MFI = 837.5) was assumed as the cut off value for the positive signal. We synthesized two additional wild-type oligoprobes (K70K-AAA-2 and T215T-ACC-2), since the original oligobeads (K70K-AAA-1 and T215T-ACC-1) gave marginal signals in some specimens (Table 1 and Fig. 4). By the modification of the position of the target codons and the length of flanking sequences, we could obtain higher MFI signals. Successful determination of the genotypes was 62/74 (83.8%), 43/74 (58.1%), 70/74 (94.6%), 55/73 (75.3%), 63/73 (86.3%) and 68/73 (93.2%) for M41, K65, K70, K103, M184 and T215, respectively. The median of background signal (without sample) was MFI = 178, and median negative signal from the patients sample was MFI = 181 (interquartile range (IQR) = 101–307). PCR-SSOP-Luminex assays create very low background and non-specific signal from negative samples. When the genotype was successfully determined by the PCR-SSOP-Luminex assay, the results were always concordant with those of sequencing (Table 2). We inferred that the failure to determine the genotype was due to sequence diversities. Therefore, we decided to customize the assay according to the sequences around K65 which had the lowest success rate (58.1%).

### Customization of oligoprobes to detect genetic diversity around K65

In 31 specimens without signals, the sequence around K65 was very diverse, especially at codons K65, K66 and K67 (Table 3). The nucleotide position of A2723G, A2747G and C2750T were frequent polymorphism for the wild type amino acid K65, K66 and D67, respectively. Fourteen specimens had a G2748A mutation that led to D67N, a thymidine analogue mutation [19]. Based on these results we synthesized 10 additional oligoprobes (Table 4).

**Table 3:** Sequence diversity around K65 in the 31 specimens.

Amino acid (HXB2-wt)	I63 K64 K65 K66 D67 S68	
Nucleotide (HXB2-wt)	ATA AAG AAA AAA GAC AGT	Number of specimens
	2747	
K66K-AAG	--- --- --- —G --- —	10
	2747 2748	
K66K-AAG/D67N-AAC	--- --- --- —G A— —	5
	2748	
D67N-AAC	--- --- --- --- A— —	3
	2750	
D67D-GAT	--- --- --- --- —T —	1
	2748	
K65K-AAG/D67N-AAC	--- --- —G --- A— —	2
	2748	
K65K-AAR/D67N-AAC	--- --- —R --- A— —a	1
	2747 2748	
K65K-AAG/K66K-AAG/D67N-AAC	--- --- —G —G A— —	1
	2741 2748	
K65K-AAG/D67N-AAC/K64K-AAA	--- —A —G --- A— —	1
	2747 2750	
K66K-AAG/D67D-GAT	--- --- --- —G —T —	1
	2748 2750	
D67N-AAT	--- --- --- --- A-T —	1
	2750 2751	
D67D-GAT/S68G-GGT	--- --- --- --- —T G-	2
		
	2747	
K65R-AGA/K66K-AAG	--- --- -G- —G --- —	1
	2750	
K65R-AGA/D67D-GAT	--- --- -G- --- —T —	2

a R: Mixed base of A and G.

**Table 4:** Design of additional oligoprobes based on clinical samples.

Locus	Name of probe	Oligoprobes	Nucleotide sequence (5'-3')
K65			ATA AAG AAA AAA GAC AGT ACT
	K66K-AAG	ATAAAGAAAAAGGACAG	--- --- --- —G --- —
	K65R-AGA/K66K-AAG	ATAAAGAGAAAGGACAG	--- --- -G- —G --- —
	K66K-AAG/D67N-AAC	ATAAAGAAAAAGAACAG	--- --- --- —G A— ---
	K65R-AGA/K66K-AAG/D67N-AAC	ATAAAGAGAAAGAACAG	--- --- -G- —G A— —
	D67N-AAC	ATAAAGAAAAAAAACAGT	--- --- --- --- A— ---
	K65R-AGA/D67N-AAC	ATAAAGAGAAAAAACAG	--- --- -G- --- A— ---
	D67D-GAT	ATAAAGAAAAAAGATAGT	--- --- --- --- —T ---
	K65R-AGA/D67D-GAT	ATAAAGAGAAAAGATAGT	--- --- -G- --- —T ---
	K65K-AAG	ATAAAGAAGAAAGACAG	--- --- —G --- --- —
	K65K-AAG/D67N-AAC	ATAAAGAAGAAAAACAGTA	--- --- —G --- A— --- -

**Figure 5:**
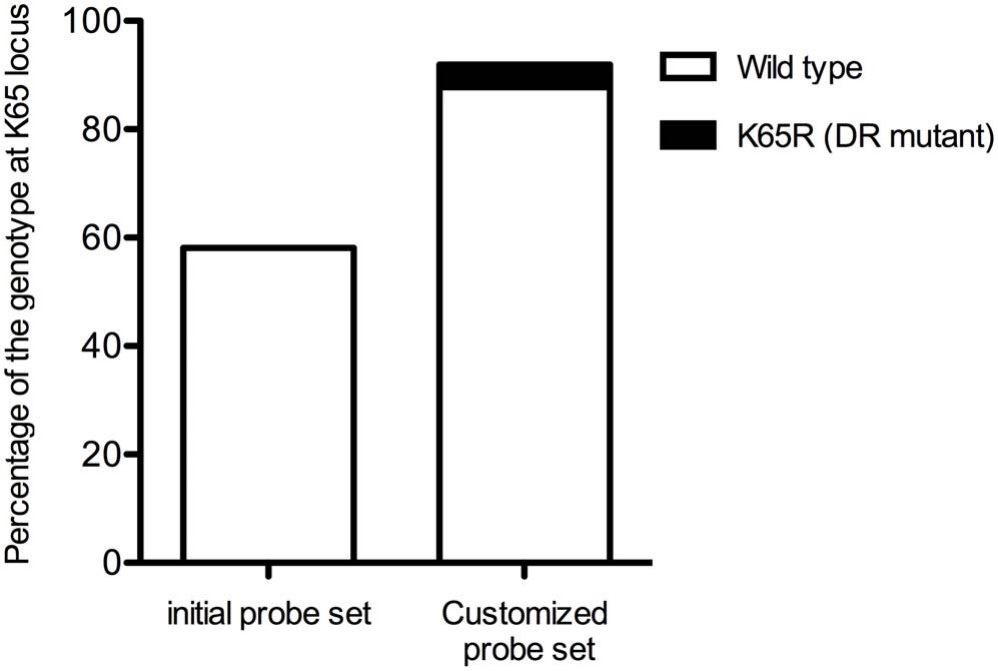
Improvement of the detection by the additional probes at K65 locus. The ordinate shows the percentage of the genotype at K65 locus determined by the PCR-SSOP-Luminex DR assay. One hundred % is the sample number (74) successfully amplified by PCR.

The specificity of the newly added oligoprobes was confirmed by the plasmids carrying the mutation (data not shown). Customization of the assay decreased the number of specimens without signals from 31 to 6 (Fig. 5).

## Discussion

We developed a PCR-SSOP-Luminex DR assay that can identify 6 clinically important DR mutations for NRTI and NNRTI in a single well. To simplify the development, we chose clade B virus and focused on the following mutations in the RT region: M41L, K65R, K70R, K103N, M184V and T215Y/F. We designed a series of capture probes according to the database of the Japanese patients. MFI from hybridization with the corresponding probes was greater than 20-fold signal-to-noise ratio in plasmid experiments (Fig. 2), at least 2-fold signal-to-noise ratio using clinical samples (Fig. 4). The initial positive reaction was as low as 58.1% (43/74) in the highly polymorphic K65 region. The use of additional probes designed to match sequences in the patients’ specimens improved the detection rate to 91.9% (68/74), demonstrating that the PCR-SSOP-Luminex assay can be customized to reflect sequences of the viruses prevalent in a given environment.

Transmission of viral strains with major DR mutations can reduce the efficacy of first-line regimens. Since the first report of a horizontal transmission of HIV-1 harboring a zidovudine-resistant mutation [20], 5% to 15% of treatment-naïve, HIV-1-infected individuals harbored the viruses with DR mutations in early 2000s in resource-rich settings [21]–[24]. It was suggested that transmission of drug resistance in the resource-rich settings can remain stable and at a low level [25]. However, in Japan where the transmission of drug-resistant viruses has historically been low, there seems to be an increasing trend [18], [26]. Rates of transmitted HIV drug resistance has remained limited also in resource-limited settings (http://www.who.int/hiv/topics/drugresistance/en/), however, limitation on the first line and subsequent regimens would be a concern. Continued surveillance of drug resistant HIV-1 is warranted.

There are some multiplex strategies to detect the single-nucleotide differences, LigAmp assay [27], [28], Nanostring assays [29], oligonucleotide ligation assay-based DNA chip [30], AS-PCR [31], [32]. These assays provide substantial improvements in their detection sensitivity over conventional sequencing-based assays, however major limitation of these assays could be detecting one or few DR mutations at a time. PCR-SSOP-Liminex assay should be able to accommodate more DR mutations than the others.

One limitation of PCR-SSOP-Luminex assay is the assay sensitivity caused by diversity of HIV. We were able to detect DR mutations that constituted 10% of the mixture in the isogenic system using plasmids. Even after successful amplification, we could not get signal in considerable number of patient’s specimens. By comparison with cloning and sequencing, we estimate that at least 20% mutant was necessary in the patient’s specimens to be detected by the PCR-SSOP-Luminex assay. The sensitivity of detection by Sanger sequencing has been reported to be ∼20% [33], [34]. Sanger sequencing and PCR-SSOP-Luminex had approximately the same detection sensitivity on patient materials. In Sanger sequencing, nucleotide sequences are determined by the wave height. When the virus in the plasma is a mixture of the wild type and a mutant, each nucleotide is displayed as two waves in the same locus with different height according to the fraction in the sample. In the case of PCR-SSOP-Luminex, the wild type or mutant nucleotides are detected independently by the signal bound to the proper probes. Therefore, as we showed partly in this paper, the mutant detection by PCR-SSOP-Luminex assay could be improved by further customization to the circulating viruses. In our paper, we used maximum 23 oligoprobes in one tube or well. The evaluation of assay volume etc. would be necessary to determine actually possible maximum number of ologibrobes in one tube. 100 color-coded beads are available, theoretically, 100 probes could be applied (http://www.luminexcorp.com/Products/Instruments/Luminex100200/). Since the wild type or mutant nucleotides are detected as the signal of hybridized probes for each in the PCR-SSOP-Luminex assay, inclusion of multiplex probes based on the codon usages for the DR mutations and the sequence variations in the flanking region of circulating viruses would improve the detection. According to our results, we could develop, validate and customized PCR-SSOP-Luminex assay for detecting DR mutations at six positions in HIV-1 RT gene. The study numbers are very limited and we worked only on subtype B HIV-1 in this article. HIV-1 diversity is notoriously huge and sequences in an individual could be more diverse than acutely expanding viruses in the field [35]. Diagnostic use in individual patients is far from the actual application at present. We believe the assay would be more suitable for molecular epidemiological studies detecting regional trends of HIV-1 DR mutation over time.

PCR-SSOP-Luminex assay were widely used for the detection of papillomavirus, influenza surveillance etc. in developing countries [36], [37]. Furthermore, application of Luminex technology to other pathogen (*C. difficile*, *Norovirus*, *E. coli* or *Salmonella*, *Rotavirus A*, *Campylobacter*, *Shigella* etc.) were reported recently [38]. It would be feasible to expect that the use of Luminex technology will be wide-spread in the near future. Furthermore, the application of PCR-SSOP-Luminex DR assay to non-clade B HIV-1 is currently under development. Future studies include refinement of the assay for use with specimens co-infected with HIV-1 and HBV.

## Conclusions

We have developed a rapid high-throughput assay for DR testing. The assay can be customized by adding oligoprobes suitable for the circulating viruses. The assay may turn out to be a useful method especially for public health research in both resource-rich and resource-limited settings.
